# Psychometric properties of the exercise addiction inventory (EAI) questionnaire among physically active young adults

**DOI:** 10.1186/s13104-024-06722-x

**Published:** 2024-03-14

**Authors:** Sahar Khoshro, Mahdieh Abbasalizad Farhangi, Leila Jahangiry

**Affiliations:** 1 Department of Community Nutrition, Faculty of Nutrition, Tabriz University of Medical Sciences, Tabriz, Iran; 2 Tabriz Health Services Management Research Center, Tabriz University of Medical Sciences, Tabriz, Iran; 3 Department of Health Education and Promotion, Faculty of Health, Tabriz University of Medical Sciences, Tabriz, Iran

**Keywords:** Exercise Addiction Inventory, Psychometric properties, Validity, Reliability, Iran

## Abstract

**Background:**

Exercise addiction (EA) is a pathological behavior pattern in which a person loses control over his exercise habits and exercises excessively and suffers negative consequences for his health and even his social life. One of the best tools to measure EA is EAI. The aim of this study was to examine the psychometric properties of the EAI among Iranian physically active young adults.

**Methods:**

Participants were randomly selected from sports clubs in Tabriz, Iran. A total of 200 questionnaires were completed. Exploratory factor analysis (EFA) and confirmatory factor analysis (CFA) were conducted along with tests of convergent validity. Reliability was assessed using Cronbach’s alpha and test-retest methods.

**Results:**

EFA indicated a one-factor structure explaining 40.43% of variance. CFA confirmed the one-factor model with good fit (Root Mean Square Error of Approximation [RMSEA] = 0.076; Comparative Fit Index [CFI] = 0.968). Internal consistency was acceptable (Cronbach’s alpha = 0.71) and convergent validity was adequate. Test-retest reliability was also adequate (intraclass correlation coefficient [ICC] = 0.71).

**Conclusion:**

The results of our study show that the Persian EAI has satisfactory psychometric properties and can be used as a valid tool to assess EA.

## Introduction

Regular exercise is defined as a set of structured, planned and repetitive complex movement activities that are performed with sufficient intensity, frequency and duration [1, 2]. Studies have shown that regular exercise may help to promote health condition and prevent some diseases, including improvements in body function, advances flexibility, lowers the risk of depression, reduced risk of lower-back problems and decreases the occurrence of type 2 diabetes mellitus and cardiovascular disease [3–5]. Despite the positive effects of exercise, in some people, excessive exercise can have harmful consequences, such as skeletal and muscular damage and immediate changes in mood [6, 7]. For the first time, the term “Exercise Addiction” (EI) was used in 1970 by Baekeland [8]. EI has been described as a “morbid pattern of behavior in which the habitually exercising individual loses control over his or her exercise habits and acts compulsively, exhibits dependence, and experiences negative consequences to health as well as in his or her social and professional life” [9]. A wide range (between 0.3 and 52) of the prevalence of EI has been reported, higher estimates from potentially “at risk” groups such as triathletes and gym goers, and lower estimates typically from the general population [10, 11]. In addition to the different sampling population, there are several other reasons for the widespread prevalence of EI, including variations in measurement scales, differences in the subjective interpretation of questionnaire items between women and men, as well as in different cultures, and intense involvement with sports is may impress the actual score of the instrument that used [6, 12, 13]. Several screening criteria have been developed to assess the risk of EA, and several questionnaires have been used to determine it [14]. The most common tools available are ‘Obligatory Exercise Questionnaire’ (OEQ) [15, 16], the ‘Exercise Dependence Scale’ (EDS) [17, 18], the ‘Exercise Dependence Questionnaire’ [19], and the ‘Exercise Addiction Inventory’ (EAI) [20]. EAI is a very short practical questionnaire with 6 items that assesses six common symptoms of addictive behaviors (i.e., conflict, salience, tolerance, mood modification, relapse, and withdrawal symptoms) aimed at identifying the risk of exercise addiction. The EAI exhibits robust internal consistency and converges with the widely used Exercise Dependence Questionnaire, evidencing its validity [20–22]. In addition to strong reliability and validity, the EAI has a solid theoretical grounding in addiction science, with clear cutoff scores for determining exercise addiction. Also, the EAI is considerably shorter than other similar questionnaires, reducing participant burden [6]. The EAI has been translated and validated for use in countries like Brazil [23], Mexico [24], Denmark [25], Italy [26] and Spain [27]. The Mexican study [24] validated the EAI in a sample of 487 regular exercisers and athletes of ages 18–52 years. The one-dimensional factor structure and adequate reliability were confirmed. Meanwhile, the Brazilian study [23] demonstrated adequate model fit, internal consistency and temporal stability in 251 university students aged around 22 years. This demonstrates its validity across different languages and cultures.

In the previous study [28] that was conducted in the Iranian population, the participants were general population, but in the current study, the participants are active young adults, and the test–retest reliability, which was not done in the previous study, is checked in this study, and the sampling method, unlike the previous study, which was convenient, is random sampling. So, the aim of this study was to examine the psychometric properties of the EAI among Iranian physically active young adults.

## Methods

### Participants and study design

In the present validity study, a three-stage cluster and random sampling strategy was utilized to recruit 200 participants (49% males vs. 51% females) aged 18–35 years (mean age: 23.1 ± 3.81 years) from sports clubs in Tabriz, Iran, from 27 May to 5 July 2022. The first stage compiled a comprehensive list of eligible clubs across 5 districts of Tabriz. In the second stage, 3 clubs were randomly selected from each district’s list, resulting in 15 total clubs. Finally, simple random sampling was used to select individual participants from the membership roster of these 15 clubs. Study purposes were explained to them. By taking part in the study, they consented to its scientific use. A further assurance was provided that all participation would remain anonymous We excluded all participants with chronic diseases affecting food intake, such as digestive problems, anorexia, and psychological problems identified by a psychologist. All participants were physically active for at least 4 h per week.

### The study questionnaire

The EAI was developed by Terry et al. in 2004 [20], and its validity and reliability were approved. EAI is a 6-item instrument. Each statement is scored on a five-point Likert scale and high ratings reflected attributes of addictive exercise behavior: 1 = Strongly disagree, 2 = Disagree, 3 = Neither Agree nor Disagree, 4 = Agree, 5 = Strongly Agree. A cut-off point of 24 indicates people at risk of exercise addiction. A score of 13–23 was considered a person with some symptoms and a score of 6–12 was considered a person without symptoms.

### Translation

The Persian translation of the questionnaire was conducted following the internationally recommended methodology developed by Beaton et al. [29].The questionnaire was backward translated to protect it from cultural influences in the context of its implementation [30]. Translating the scale to Persian was performed by two bilingual health professionals. An aggregated version was then developed for use in the survey. In case of differences between the two translated versions, this issue was resolved through discussions with the translators to achieve a provisional unified translation. For significant disagreements, a third independent translator was recruited to conduct further checks. In the next step, two English translators without prior knowledge of the questionnaire reviewed and translated it into English to compare and evaluate with the original English version and ensure that there were no discrepancies.

### Face and content validity

A qualitative face validity test was conducted on the questionnaire. A sample of physically active young adults (*n* = 10) was asked to evaluate the tool and give feedback for improvement. The scale was modified as a result of this process. EAI’s provisional Persian model was evaluated by an expert panel. The appropriateness and relevance of items to Iranian youth and their cultural context were reviewed by 10 professors in nutrition, health, and sports physiology. Content Validity Index (CVI) and Content Validity Ratio (CVR) were calculated, respectively, based on three indicators including clarity, relevance and simplicity and based on the “necessity” index. The content validity of each item was confirmed by ensuring that its CVR (Content Validity Ratio) was greater than 0.62, as per the Lawshe Table [31]. Items with a CVI greater than 0.79 were deemed suitable. To assess face validity and to improve clarity, the pre-final version of the questionnaire was reviewed by 10 eligible young adults. Because none of the questions were deleted, the length of the Persian EAI model was equal to the original questionnaire.

### Sample size considerations

According to the subject-to-item ratio method, it is recommended to have a range of 2 to 20 individuals per item for exploratory factor analysis (EFA) [32]. Additionally, a minimum sample size of 100 to 250 is suggested. Similarly, literature suggests a range of 150 to 1000 participants for Confirmatory factor analysis (CFA) [32]. In this study, we have a total of 6 items in the questionnaire. Therefore, a minimum sample size of 60 individuals (10 times the number of items) was considered necessary. However, to ensure compliance with the minimum sample size, the researchers decided to increase the sample size to 200 participants.

### Statistical analysis

SPSS version 25 and Amos 24.0 were used for data analysis. To evaluate the sampling adequacy of factor analysis, Kaiser-Meyer-Olkin (KMO) and Bartlett’s test of sphericity were used. For factor extraction, any factor with an eigenvalue equal to one or higher was considered significant. For extraction in factor analysis, principal component analysis with varimax rotation was used if the loading criterion was 0.4 or more. CFA was conducted on the one-factor model derived from the EFA, employing the maximum likelihood estimation method. Additionally, various model fit indices were assessed, including χ2/df, Goodness-of-Fit Index (GFI), Adjusted Goodness-of-Fit Index (AGFI), Comparative Fit Index (CFI), Tucker-Lewis Index (TLI), Root Mean Square Error of Approximation (RMSEA), and Normed Fit Index (NFI). Acceptable values are as follows: values between 2 and 5 for $\chi 2/df$; > 0.90 for GFI, NFI, CFI, and TLI; > 0.85 for AGFI; and between 0.05 and 0.080 for RMSEA.

### Data collection

We collected the demographic variables including age, gender, weight, height, body mass index (BMI), education level, and marital status. We also used the International Physical Activity Questionnaire-Short Form (IPAQ-SF) to evaluate physical activity.

## Results

### Construct validity

**Fig. 1:**
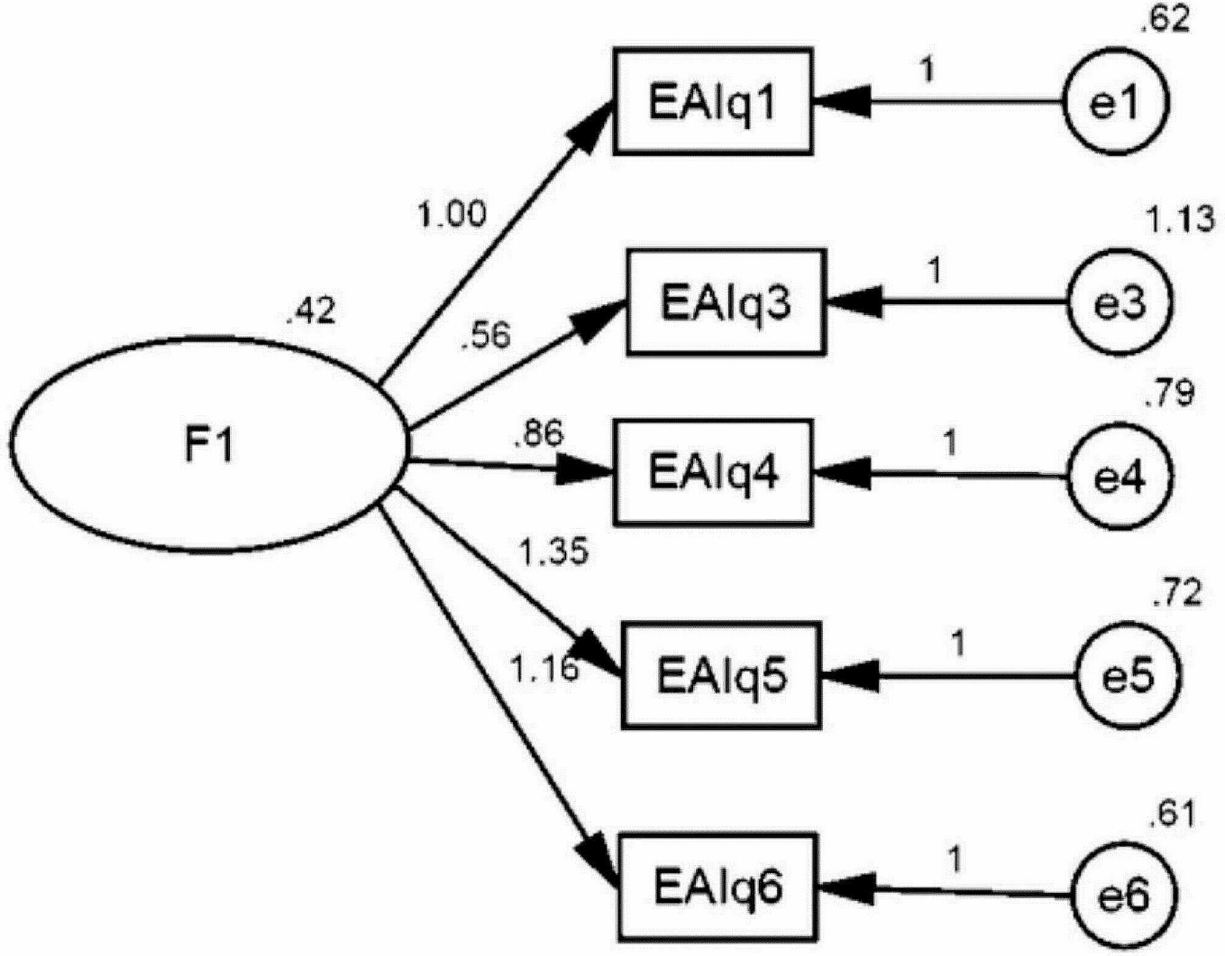
A one-factor model for the scale gained from the confirmatory factor analysis (*n* = 200)

The KMO was 0.732; (*P* < 0.0001), and Bartlett’s test of sphericity was significant (χ2 = 189/281, *p* < 0.0001), which indicated the adequacy of the sample for factor analysis. Principal Component Analysis (PCA) and Varimax rotation were used to extract factors. To maintain consistency with the original version, we have set the number of factors at 1. One -factor solution explained the 40.43% variance. In Table 1, factors and their factor loadings are summarized. We ignored the item2 that had a factor load lower than 0.4 [33]. We conducted a CFA on the Persian EAI to evaluate the fitness of the model obtained from the EFA. The fit of the model is shown in Fig. 1. All goodness-of-fit indices of CFA unequivocally validated the suitability of the model (${\chi }^{2}/df$ = 2.145; RMSEA = 0.076 (90% CI = 0.001–0.139); GFI = 0.980; AGFI = 0.939; NFI = 0.943; CFI = 0.968; TLI = 0.936).

**Table 1:** Results of Factor Loadings for the EAI

Items	Factor 1	Factor 2
6/If I cut down the amount of exercise I do, and then start again, I always end up exercising as often as I did before	0.769	
5/If I have to miss an exercise session, I feel moody and irritable	0.759	
1/I use exercise as a way of changing my mood	0.725	
4/Over time I have increased the amount of exercise I do in a day	0.683	
2/Conflicts have arisen between me and my family and/or my partner about the amount of exercise I do		0.839
3/I use exercise as a way of changing my mood		0.607

EAI Exercise Addiction Inventory; * Factor loading higher than 0.4 is acceptable

### Reliability and convergent validity

We used Cronbach’s alpha to measure internal consistency. The Cronbach’s alpha coefficient for the EAI was 0.71 that was acceptable. To evaluate the stability of the EAI scale, test-retest analysis was used. The results were acceptable. The intra-class correlation (ICC) was 0.71 (95% CI = 0.64–0.77).

Reliability was also assessed through the calculation of composite reliability (CR) and maximum reliability (MaxR or H) [34]. To evaluate convergent validity, the average variance extracted (AVE) was calculated and compared with the CR. The values of CR, and MaxR(H) were 0.68, and 0.76, respectively, indicating acceptable reliability [34, 35]. AVE was 0.35 and below of the cut-off (i.e. 0.5). According to Fornell and Larcker [36], if the Average Variance Extracted (AVE) is less than 0.5 and the Composite Reliability (CR) is higher than 0.6, the construct’s convergent validity is still considered acceptable.

### Demographic findings of participants

Out of 200 participants, 98 (49%) were men and 102 (51%) were women, and their average age was 23.1 ± 3.81. Among these participants, 28 subjects (14%) had a diploma, 3 (1.5%) had an associate degree, 124 (62%) had a bachelor’s degree, 38 (19%) had a master’s degree, and 7 (3.5%) had a Ph.D. Also, 183 subjects (91.5%) were single and 17 (8.5%) were married. The total score of EAI was higher in the age range of 18–20 years compared to 20–35 years old and also among subjects with normal BMI compared to others. Furthermore, this score was higher among people those who exercised with high intensity compared to than those who exercised with low to moderate intensity. As shown in Table 2.

**Table 2:** Descriptive report of participants’ means score and total score of exercise addiction inventory domains (*n* = 200)

Variables		N (%)	Total score
**Age**	18–20	46 (23)	16.23 (4.15)
	20–30	142 (71)	15.69 (3.69)
	30–35	12 (6)	15.50 (2.68)
**Gender**	Male	92 (49)	15.81 (3.74)
	Female	102 (51)	15.51 (3.55)
**Marital status**	Single	183 (91.5)	17.69 (4.77)
	Married	17 (8.5)	18.58 (4.43)
**Education**	Diploma	28 (14)	16.50 (3.63)
	Associate degree	3 (1.5)	18.33 (2.08)
	Bachelor’s degree	124 (62)	15.77 (3.91)
	Master’s degree	38 (19)	15.10 (3.56)
	Ph.D.	7 (3.5)	16.57 (1.99)
**BMI**	Low	9 (4.5)	15.33 (2.73)
	Normal	132 (66)	15.99 (3.52)
	High	49 (24.5)	15.55 (4.39)
	Obese	10 (5)	15.10 (4.17)
**Physical activity**	Low	54 (27)	14.27 (3.36)
	Moderate	38 (19)	15.13 (2.99)
	High	108 (54)	16.81 (3.87)

BMI, body mass index

## Discussion

The present study aimed to assess the psychometric properties of the Persian version of the EAI. The results of exploratory factor analysis showed a one-factor structure explaining 40.43% of the variance and is consistent with previous studies in this field [23–25]. CFA confirmed the one-factor model with acceptable fit indices.

In this study, Cronbach’s alpha of 0.71 indicates acceptable internal consistency, although slightly lower than the original EAI (0.84) [20] and other adapted versions (> 0.80) [23, 24]. The 2-week test-retest reliability (ICC = 0.71) was adequate [23, 25]. Overall, the Persian EAI demonstrates satisfactory reliability.

In contrast to previous EAI validation studies that supported a 6-item single factor structure (24, 37), EFA in the current study resulted in a 5-item Persian EAI model. Unlike previous research conducted in general population samples in Iran [28], the present study focused specifically on physically active young adults. The specificity of sampling only regular exercisers may have contributed to the discrepant factor structure obtained here compared to past studies with more diverse participants. Additionally, the current study utilized in-person interviews for data collection, while past Iranian research relied on online surveys [28]. The smaller sample size achieved here (*n* = 200) relative to larger online samples may have also impacted results.

The EAI score of women was lower than men in the present study. A number of previous studies have reported similar findings [37, 38] but some disagree [39, 40]. In studies that include eating disorders, women score higher on EA scales [12, 41]. A better understanding of the gender differences in risk for EA should consider whether eating disorders are present or absent.

A limitation of this study was that concurrent validity of the Persian EAI could not be evaluated, since no gold standard exercise addiction questionnaire was available in Persian for comparison.

## Conclusion

The results of the present study showed that the Persian EAI has acceptable validity and reliability for evaluating EA. It is a simple and practical tool that can be used for future studies. For most people, exercise has beneficial effects and is widely recommended. However, diagnosing exercise addiction can be complex, and the EAI can quickly assess the risk of exercise addiction.

## Data Availability

Data are available with reasonable request from the corresponding author.
